# Iron Metallodrugs: Stability, Redox Activity and Toxicity against *Artemia salina*

**DOI:** 10.1371/journal.pone.0121997

**Published:** 2015-04-07

**Authors:** Hector Aguilar Vitorino, Luca Mantovanelli, Flavia Pinheiro Zanotto, Breno Pannia Espósito

**Affiliations:** 1 Instituto de Química, Universidade de São Paulo, São Paulo, Brazil; 2 Instituto de Biociências, Universidade de São Paulo, São Paulo, Brazil; CINVESTAV-IPN, MEXICO

## Abstract

Iron metallodrugs comprise mineral supplements, anti-hypertensive agents and, more recently, magnetic nanomaterials, with both therapeutic and diagnostic roles. As biologically-active metal compounds, concern has been raised regarding the impact of these compounds when emitted to the environment and associated ecotoxicological effects for the fauna. In this work we assessed the relative stability of several iron compounds (supplements based on glucoheptonate, dextran or glycinate, as well as 3,5,5-trimethylhexanoyl (TMH) derivatives of ferrocene) against high affinity models of biological binding, calcein and aprotransferrin, via a fluorimetric method. Also, the redox-activity of each compound was determined in a physiologically relevant medium. Toxicity toward *Artemia salina* at different developmental stages was measured, as well as the amount of lipid peroxidation. Our results show that polymer-coated iron metallodrugs are stable, non-redox-active and non-toxic at the concentrations studied (up to 300 µM). However, TMH derivatives of ferrocene were less stable and more redox-active than the parent compound, and TMH-ferrocene displayed toxicity and lipid peroxidation to *A*. *salina*, unlike the other compounds. Our results indicate that iron metallodrugs based on polymer coating do not present direct toxicity at low levels of emission; however other iron species (eg. metallocenes), may be deleterious for aquatic organisms. We suggest that ecotoxicity depends more on metal speciation than on the total amount of metal present in the metallodrugs. Future studies with discarded metallodrugs should consider the chemical speciation of the metal present in the composition of the drug.

## Introduction

Metallodrugs are either organometallic or coordination compounds designed to have pharmacological activity. Although several metal derivatives have long been used to treat diseases such as arthritis, obsessive-compulsive disorder, stomach ulcer and microbial infestations, it was only after the advent of cisplatin, used for the treatment of cancer in the 1960’s, that metal-based compounds with medicinal properties received systematic attention [1]. More often than not, the association of a metal ion to an organic molecule, i.e. a metallodrug, can lead to synergistic activity that may modulate either the effect of the metal or the properties of the organic molecule [2].

Approved iron metallodrugs include human or veterinary supplements based on the association of iron to a sugar- or amino acid-based coating. Also, the anti-hypertensive agent sodium nitroprussiate is an iron-based compound. Candidate iron metallodrugs are the ferrocene derivatives such as 3,5,5-trimethylhexanoyl (TMH)-ferrocene and ferrochloroquine, with interesting antitumoral, antimalarial, antifungal and antibacterial activities [3–5]. Even though iron is an essential nutrient, the use of iron metallodrugs is not without risks. Indeed, iron overload and subsequent oxidative damage after the use of iron supplements [6] or ferrocene derivatives [7–10] have been reported in the literature.

From an environmental perspective, the levels of both metal derivatives [11] and pharmaceutical waste [12] have increased due to anthropogenic activity, accidental spills, and lack of control or regulations. Metallodrugs may be especially hazardous as they are designed for high biological activity and they carry a metal ion as a non-biodegradable core. Accidental exposure to excess of iron metallodrugs could be even more deleterious, since organisms lack the ability to handle iron as a potentially toxic element, as is the case for other metals such as mercury and lead. Iron toxicity could be restricted to undesirable effects for organisms (oxidative stress following iron overload) and/or could affect the ecosystem (water eutrophication).

As environmental chemistry information is lacking for iron metallodrugs, in this work we studied the aqueous stability and redox-active properties of two different classes of derivatives (polymer-coated and organometallic iron compounds), followed by the study of the mechanisms of toxicity to *Artemia salina* (Linnaeus, 1758) (Crustacea, Artemiidae), chosen as a model of ecotoxicological impact. *Artemia* has been used as an ecotoxicological model for both inorganic nanomaterials in general [13–15] and for iron complexes studies [16–18].

## Materials and Methods

### Iron metallodrugs

Eight iron supplements with different organic coatings for the iron hydroxide core, for both human and veterinary use, were obtained from laboratories in Brazil. They consisted in the dextran derivatives Biovet (Biovet), Dexiron (FATEC), Ferro dextrano (Uzinas Chimicas Brasileiras), Fertal (Mogivet), Ferrodex (Tortuga), and also in Gleptoferril (iron glucoheptonate; Eurofarma), Neutrofer (iron glycinate, EMS Sigma Pharma) and Noripurum (iron maltose; Nycomed). All drugs were marketed as containing 100 mg/mL total iron concentration (except for Neutrofer and Noripurum, which were 50 mg/mL). The iron concentration of each sample was confirmed by furnace atomic absorption spectrometry in a Shimadzu AA-6300 equipment.

**Fig 1:**
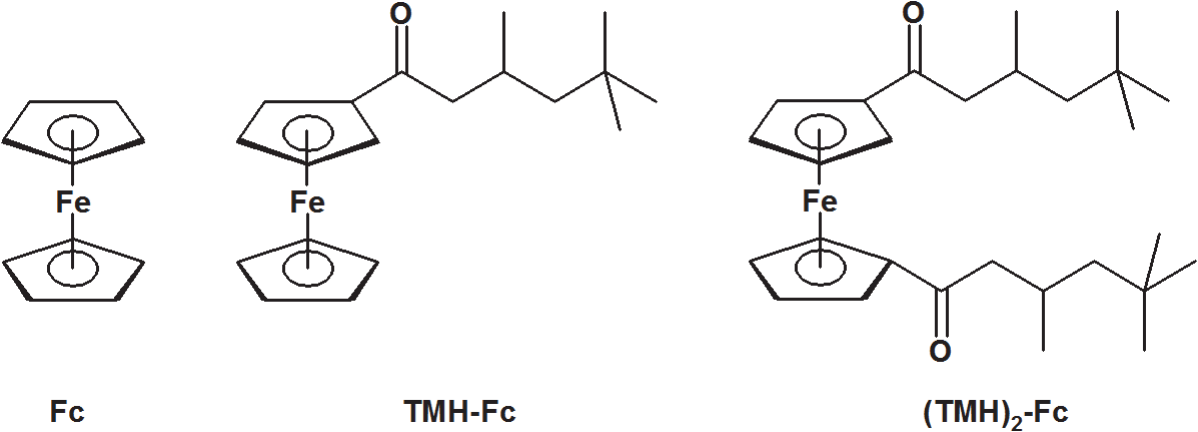
Metallocenes under study.

Ferrocene (Fc) and the reagents used in the preparation of its 3,5,5-trymethylhexanoyl (TMH) derivatives (Fig 1) were procured from Sigma-Aldrich. TMH-Fc and (TMH)_2_-Fc were obtained by means of a described protocol [19] with modifications. Reactions were carried out in round flasks under N_2_ atmosphere and magnetic stirring. TMH-Fc: a solution of AlCl_3_ (1.3 g; 9.7 mmol) in 20 mL dichloromethane was mixed with 20 mL of dichloromethane containing 1.5 g (8.1 mmol) Fc. After, 1.4 g (8.1 mmol) TMH chloride was added dropwise and the system was kept in cold water (~ 3^°^C). (TMH)_2_-Fc: a solution of AlCl_3_ (1.8 g; 13 mmol) in 20 mL dichloromethane was mixed with 20 mL of dichloromethane containing 2.2 g (12 mmol) TMH chloride. After, a solution of 1.0 g (5.4 mmol) Fc in 20 mL dichloromethane was added dropwise and the system was kept in cold water (~ 3^°^C).

The mixtures above were stirred at room temperature for 24 h, and then 50 g of ice were added. The organic phases were separated and concentrated by rotative evaporation. The oil residues were dissolved in 10 mL toluene and separated in a silica gel column (20 × 1 cm) eluted with this solvent. Eluates were analyzed by means of thin layer chromatography. Fractions containing TMH-Fc (R_f_ 0.43) or (TMH)_2_-Fc (R_f_ = 0.26) were pooled and dried. The oily residues were redisolved in ethanol and the filtrates were dried at low pressure, resulting in reddish-brown oil products.

For TMH-Fc: Yield = 59% (1.6 g). Elemental analysis (C_19_H_26_OFe, 326.3 g.mol^-1^): calculated (%), Fe = 17.12; C = 69.95; H = 8.03. Experimental (%), Fe = 17.22; C = 69.40; H = 7.96. Infrared peaks (*v*_max;_ cm^-1^): 3098, 2955, 2904, 2868, 1669, 1454, 1378, 1277, 1028, 482. ^1^H NMR (300 MHz, CDCl_3_): δ 4.20 (s, 4 aromatic H), δ 4.48–4.50 (t, 2 aromatic H), δ 4.77 (d, 2 aromatic H), δ 2.50–2.73 (dd, 2 methylene groups), δ 2.23–2.25 (m, main chain H), δ 1.00–1.03 (d, main chain H), δ 1.12–1.34 (m, 9 methylene H), δ 0.94 (m, 2 main chain H), δ 0.94 (s, 9 methylene H).

For (TMH)_2_-Fc: Yield = 54% (1.3 g). Elemental analysis (C_28_H_42_O_2_Fe, 466.5 g.mol^-1^): calculated (%), Fe = 12.0, C = 72.1; H = 9.07. Experimental (%), Fe = 10.9, C = 69.6; H = 9.41. Infrared peaks (*v*_max;_ cm^-1^): 3218, 3104, 2952, 2901, 2869, 1730, 1675, 1375, 1275, 1028, 482. ^1^H NMR (300 MHz, CDCl_3_): δ 4.49–4.51 (t, 4 aromatic H), δ 4.78 (m, 4 aromatic H), δ 2.50–2.71 (dd, 4 H main chain), δ 2.20–2.26 (m, 2 H main chain), δ 2.23–2.25 (m, H main chain), δ 1.03–1.05 (m, 6 methylene groups), δ 1.15–1.36 (m, 4 H main chain), δ 0.95 (s, 18 H main chain).

### Stability assays

Ecotoxicology models such as the Free-Ion Activity Model assume that only free (i.e., non-chelated) metal would be available for exerting any kind of biological activity against a target organism [20,21]. To verify whether or not free iron from the compounds studied here would correlate with toxicity to *Artemia salina*, stability assays were conducted in two media with different salinities (artificial seawater and physiological buffer). The amount of iron released from the iron metallodrugs was determined by means of competitive assays with the fluorescent probes calcein or fluorescein-transferrin (FlTf). Both probes display quick and stoichiometric fluorescence quenching upon iron binding [22]. FlTf was prepared by the coupling of 5-DTAF (5-(4,6-dichlorotriazinyl)aminofluorescein) to holo-transferrin as previously described [23]. In flat, transparent 96 wells microplates, aliquots of 10 μL of the iron compounds with different concentrations or 10 μL of iron standards (ferrous ammonium sulfate in aqueous solution) were treated with 190 μL of 2 μM calcein or 190 μL of 2 μM FlTf supplemented with 10 mM sodium bicarbonate. Calcein and FlTf solutions were prepared in either physiological buffer HBS (Hepes Buffered Saline, hepes 20 mM, NaCl 150 mM, Chelex 1 g/100 mL; pH 7.4) or artificial seawater (sea salt at 3.5% salinity, pH 8.3, Chelex 1 g/100 mL, filtered under low pressure with a 0.22 nm cellulose membrane). The microplate was incubated at 37^°^C in a BMG FluoStar Optima instrument and the fluorescence of the samples (λ_exc_/λ_emis_ = 485/520 nm) was registered in 1 min intervals during 24 h. Experiments were performed in eight replicates and repeated four times.

### Pro-oxidant activity of the iron compounds

The content of pro-oxidant iron in the metallodrugs under physiological conditions was assessed using the rate of dihydrorhodamine (DHR) oxidation in the presence of ascorbate [24]. In flat, transparent 96 wells microplates, 20 μL aliquots of 40 μM iron complexes in HBS (or 20 μL of ferric nitrilotriacetate standards) were treated with 180 μL of a mixture of DHR 50 μM and ascorbic acid 40 μM in HBS. Fluorescence intensity was registered at 37^°^C in a BMG FluoStar Optima instrument (λ_exc_/λ_emis_ = 485/520 nm) in 2 min intervals during 1 h. Oxidation rates (fluorescence/minute) were determined from the slope of the kinetic curves after the t = 15 min point. Experiments were performed in eight replicates and repeated 4 times.

### Toxicity to *Artemia salina*

Toxicity tests to *Artemia salina* (INVE, Artemia High 5, Brazil) were conducted with adult individuals (after approximately 8 days hatching) and with individuals at stage 1 after hatching (time zero), according to described protocols [14,25].

Toxicity to adult *Artemia*. Male adult *A*. *salina* (400 individuals) were screened and transferred to a Petri dish for acclimatization for 1 h. Artificial seawater was prepared with distilled water and sea salt (Red Sea Salt) at 3.5% salinity, pH 8.3, and filtered under low pressure with a 0.22 nm cellulose membrane (Millipore). Different volumes of the iron compounds dissolved in HBS were transferred to 40 mL flasks (25 cm^2^ tissue culture flask from TPP), and the volume was completed to 5 mL with artificial seawater to achieve concentrations between 0–1000 μM. Ten animals were then transferred to each flask and incubated at 30^°^C in the dark. After 24 h, live and dead animals were counted. This experiment was performed in triplicate.

Toxicity to stage 1 *Artemia*. *Artemia* was hatched in a tank (20×10×15 cm) using a thermostat, light source and air metering. Artificial seawater was prepared with distilled water and sea salt (Red Sea Salt) at 3.5% salinity, pH 8.3, and filtered under low pressure with a 0.22 nm cellulose membrane (Millipore). In two liters of artificial seawater, 0.5 gram of *Artemia* cysts was added and kept under light for 24 h. Then, ten animals were separated and treated with 0–300 μM of the iron compounds in the dark. Live and dead animals were counted after 24 h. This experiment was performed in quadruplicate.

*Lipid peroxidation*. The lipoperoxidation activity of the most toxic compounds was assessed by the modified Fox method as cumene hydroperoxide (CHP) equivalents [26,27]. Stage 1 *Artemia* were treated with iron compounds as described, then frozen and homogenized in 100% methanol. Homogenates were centrifuged (Eppendorf 5424R micro centrifuge) at 15000 rpm and the supernatant (15 μL) was transferred to 96 well microplates. The samples were treated with 90 μL FeSO_4_ (1 mM), 35 μL H_2_SO_4_ (0.25 M), 35 μL xilenol orange (1 mM) and 175 μL water. Incubation for 1 h was performed at room temperature, and absorption was measured at 580 nm. Then, 10 μL of CHP (175 μM) were added to the samples and absorbance at 580 nm was read again after 15 min.

Kruskal-Wallis One Way Analysis of Variance on Ranks followed by Tukey Test were performed with Stat 3.2 software.

## Results and Discussion

### Stability assays

Calcein and transferrin are both strong iron(III) chelators (log*K* = 24 [28] and 20–21 [29], respectively), therefore they are useful as surrogates of high affinity biological substrates in the environment. Also, the fluorescence of calcein and FL-Tf is stoichiometrically quenched by iron, rendering these molecules useful for detection of the metal at very low concentrations by means of competitive equilibrium assays. The underlying concept is, the higher the stability of the iron-based metallodrug, the lesser free iron will be available for coordination with either calcein or FL-Tf, and hence the lesser the fluorescence quenching of these probes. By means of a calibration curve with “free” iron, such as FAS, it is possible to quantify the amount of iron released by the iron metallodrugs and then estimate their stabilities [22].

Once emitted to the environment, iron metallodrugs can either be absorbed as intact molecules (which will dissociate within the organism), or they can dissociate, thus making the hydrolysis products responsible for an array of biological effects. Stability studies were conducted in both physiological (HBS) and seawater conditions, in order to assess both possibilities.

Polymer-coated iron metallodrugs at a [Fe]_total_ = 2 μM maximum concentration displayed in general great stability against either calcein or FlTf in neutral medium, even at non-zero ionic strength, as we were not able to detect significant fluorescence quenching of either probe upon reaction with these compounds (data not shown). Indeed, free iron arising from the dissociation of these molecules has been detected only at higher drug concentrations (200 μM in iron) and comprises usually ~3% of the total metal [6]. Our findings indicate that low levels of emissions of these drugs are not expected to induce high levels of free iron in environmental samples.

**Fig 2:**
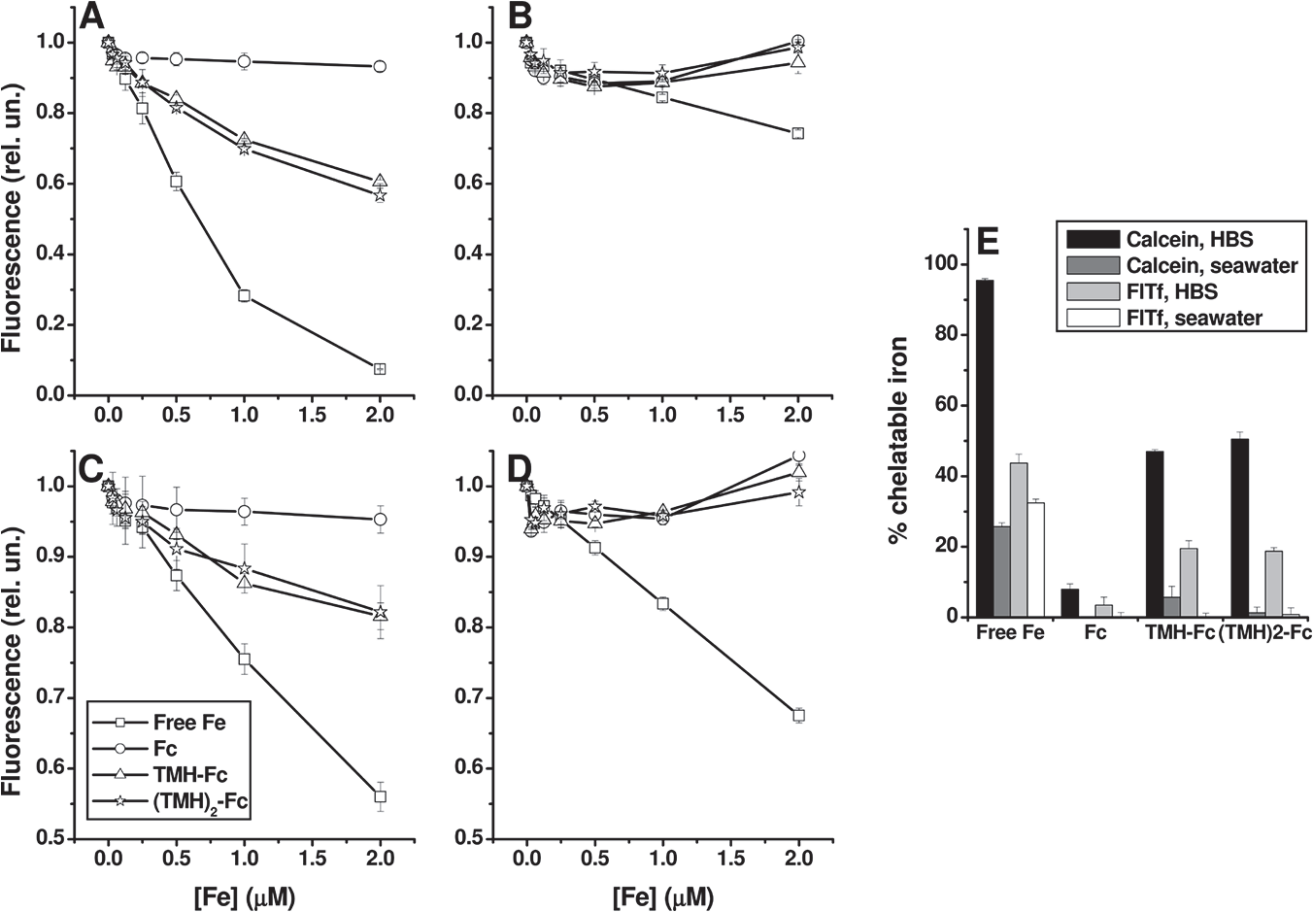
Quenching of 2 μM calcein in (A) HBS or (B) artificial seawater, and of 2 μM Fl-Tf in (C) HBS or (D) artificial seawater, caused by Fc derivatives. (E) Percentage of iron available to these probes (chelatable iron), calculated at the highest Fe concentration (2 μM). Rel. un. = relative fluorescence units.

On the other hand, candidate metallodrugs derived from the Fc framework displayed lower stability against the high affinity chelator models in the physiological buffer (Fig 2). Binding stoichiometry of the probes (ligand:iron) is 1:1 (calcein) or 1:2 (FlTf), therefore 2 μM of each probe can detect up to 2 μM (calcein) or 4 μM (FlTf) chelatable iron. At the highest iron concentration (2 μM), iron salts are virtually completely chelated, as expected. The parent metallocene is very stable, however both its TMH derivatives show ~ 40*–*50% decomposition against the probes. In these compounds, deactivation of the organometallic bonds by the carbonyl groups from the 3,5,5-trimethylhexanoyl moiety explains their relative lack of stability and expulsion of the metal from the cyclopentadienyl sandwich [30]. This decreased stability of the TMH derivatives agrees with infrared spectrometry data, where both TMH-Fc and (TMH)_2_-Fc display a Fe-C_5_ stretching band at 482 cm^-1^, while in Fc this band occurs at 460 cm^-1^, indicating a weakened bond in the TMH derivatives. These experiments were repeated for higher ferrocenyl concentrations (up to 80 μM), and the relative trend of stability was not altered (data not shown).

Ferrocene and its derivatives displayed no detectable amount of free iron in artificial seawater with any of the Fe probes. Since the salinity of artificial seawater is higher than that of physiological buffer (~0.6 against 0.15 M in NaCl, respectively), the increased amount of Cl^-^ ions may efficiently compete with the fluorescent probes for metal coordination, and although chloride forms weak complexes with Fe(III) (log*K* = 1.06 for [FeCl_2_]^+^ [31]), its concentration is 300 times higher, therefore any trace amounts of iron released from the ferrocenes may not be accessible to the probes. Fluorescent transferrin binds more iron even in high salinity medium, as the metal is accommodated in a protein fold with little exposure to the solvent [29] in a kinetically inert form.

### Pro-oxidant activity of the iron compounds

In biological fluids, labile forms of iron may participate in the auto-oxidation cycle of endogenous ligands such as ascorbate, leading to the development of a plethora of reactive oxygen species, indicating that even otherwise “antioxidants” may behave as pro-oxidants depending upon their relative proportion with redox-active metal ions. The formation of these reactive species is conveniently measured as the rate of conversion of the non-fluorescent probe dihydrorhodamine to its fluorescent counterpart [24].

In agreement with the stability assays, we found that the polymer-coated iron metallodrugs of commercial use displayed no measurable redox-active iron (values were lower than the detection limit of 0.4 μM [24]). Previous reports indeed show that only 0.8% of the total iron in similar formulations is redox-active [6]. Iron from the Fc derivatives, on the other hand, was able to engage into ROS generation mediated by ascorbate auto-oxidation, the TMH derivatives being more active than the parent metallocene (Table 1). This is in agreement with the higher amount of free iron available from the dissociation of the TMH derivatives (Fig 2). Organometallic complexes based on essential, redox-active ions are in general relatively stable, but they may cause oxidative stress. Depending upon the desired biological outcome, control of this property might be tuned in order to develop new Fc derivatives with potential biological activity [32].

**Table 1:** Concentrations (μM) and percentage of redox-active iron (%) in samples of Fc derivatives (average ± s.d. of 8 measurements).

**Iron compound**	**Redox-active Fe (**μ**M)**	**Redox-active iron (%)**
**Fc**	1.20 ± 0.17 ^a^	3.0
**TMH-Fc**	3.80 ± 0.17 ^b^	9.5
**(TMH)**_**2**_ **-Fc**	3.26 ± 0.15 ^c^	8.2

Total iron concentration = 40 μM.

^a^ (p < 0.05)

^b^ (p < 0.05)

^c^ (p < 0.05)

### Toxicity to *Artemia*

The toxicity of iron compounds with possible pharmacological activity has already been tested against *Artemia salina*. Iron complexes with thiosemicarbazones, which were proposed as antitumor and antimicrobial agents, displayed LD50 values in the range of 14–30 μM for 2-pyridineformamide derivatives [17] or 2–5 μM for *para*-tolyl derivatives [18]. On the other hand, iron magnetic nanoparticles proposed for diagnostic or drug delivery strategies have very low lethality to *Artemia* at concentrations ~ 1 mg/mL, whether or not coated with silica [13], despite decrease in swimming speed and onset of inflammation symptoms [15]. Finally, Al-Bari and colleagues observed that LD50 of four thio- or alcoxy-ferrocene derivatives against *Artemia salina* nauplii was in the range or 20–330 μM [16].

In this work, besides evaluating other candidate iron metallodrugs (ferrocenes), it was assessed, for the first time, the acute toxicity of commercial iron supplements to *A*. *salina*. Prolonged exposure (> 24 h) to the metal compounds was not conducted because the metal compounds will hydrolyze and precipitate in saline, alkaline medium.

**Fig 3:**
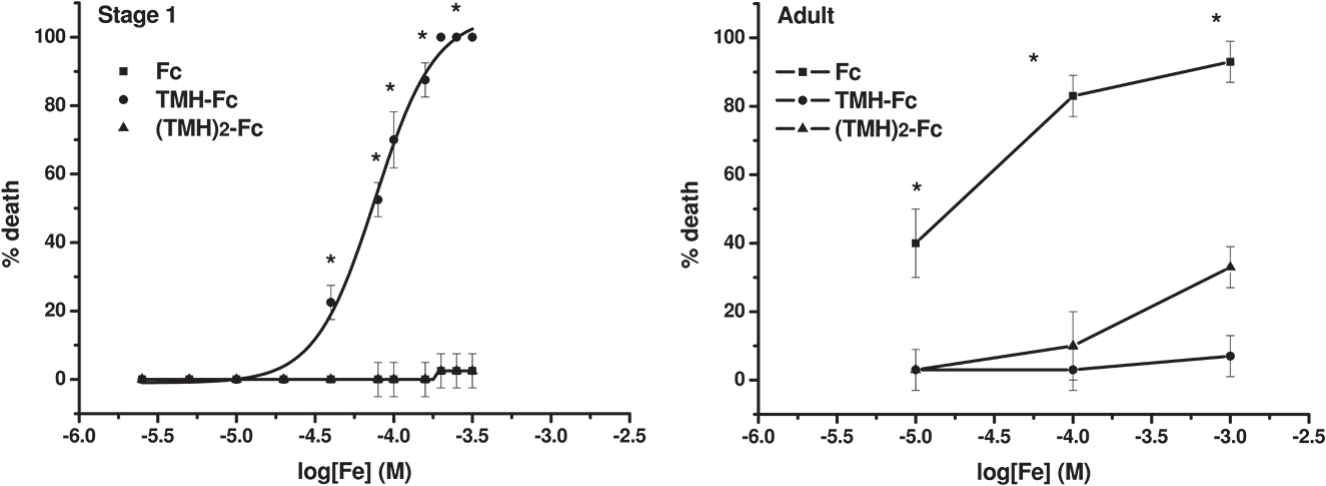
Dose-response curves for adult or stage 1 *Artemia* treated with ferrocene and its derivatives. Effects of Fc (in adults) and TMH-Fc (in stage 1 animals) were statistically different (*, P < 0.05).

“Free” iron (as FAS) caused only 37% lethality at 1000 μM final concentration in adult *Artemia*. Indeed, concentrations of free iron up to 185 μM are considered sublethal to this organism [33]. All commercial iron metallodrugs under study displayed only < 10% lethality to adult *A*. *salina* at 1000 μM final concentration (data not shown). Therefore, the polymer coatings alone (glycine, dextran, maltose, glucoheptonate) were not tested. The substituted Fc derivatives displayed toxicity similar to the polymer-coated metallodrugs, however Fc alone was lethal for 40% (at 10 μM), 90% (at 100 μM) and 100% (at 1000 μM) of the individuals (Fig 3). The toxicity of Fc does not correlate with amount of free iron, as probed by the stability assays showed here. This indicates that the whole molecule is responsible for toxic effects in adult *Artemia*.

Polymer-coated iron derivatives were also non-toxic to stage 1 *Artemia* (data not shown). The absence of toxicity of Fc (Fig 3) correlates with the virtual lack of free and/or redox-active iron (Fig 2 and Table 1), however free iron (as FAS) had only 2.5% lethality up to 300 μM. Toxicity therefore results from properties of the whole molecules rather than from simple metal release, because TMH-Fc and (TMH)_2_-Fc displayed similar stability (Fig 2) and elevated redox-active iron (Table 1). Indeed, Fc and (TMH)_2_-Fc were virtually non-toxic (< 2.5% lethality) to stage 1 *Artemia* up to 300 μM. TMH-Fc, on the other hand, displayed a distinctive dose-response behavior (Fig 3), with a LD50 of 76.6 ±1.0 μM, which is close to other candidate ferrocene metallodrugs with low polarity substitutes [16]. Our data are in agreement with previous reports on the relative absorption of these compounds using a mammalian model. For example, rats fed with 5 mg/day with Fc, TMH-Fc and (TMH)_2_-Fc displayed whole body iron retention of 24, 48 and 6%, respectively [13]. In addition, there are reports of oxidative damage to the liver [34] and astrocytes [35] of mice treated with TMH-Fc. It must be pointed out that bioavailability and absorption through any given biological interface are determined by factors such as lipophilicity and molecule size. Fc is more lipophilic than their derivatives [19], which may difficult its solubility in an aquatic environment, but also facilitate its way through biological membranes and cells. In addition, (TMH)_2_-Fc is a bigger molecule than the mono-substituted Fc, which may difficult the traffic across biological membranes.

Adults, on the other hand, were more susceptible to Fc, which was non-toxic to stage 1 animals (Fig 3). Adult *Artemia*, in general, should be less susceptible to metallodrugs than larval stages, because earlier larval stages are undergoing major physiological and morphological changes through successive short larval stages and frequent molting, rendering these earlier stages more susceptible to toxicants as a whole. However, routes of absorption are expected to change sharply as the animal develops, going from a non-functional digestive system and yolk dependent larvae up to five days after hatching, to a fully formed digestive system for adults. The routes for absorption, therefore, change from body surface from day 1 larvae to ingestion for adults. In addition, the behavior of Fc was similar to some other contaminants such as substituted phenols [36] or chlorinated solvents [37], which also are progressively more toxic to *Artemia* as the animal ages. At the moment we cannot explain these discrepancies, but results do suggest that Fc could be easily ingested by adults due to its lipophilic nature and easy access to digestive system and biological membranes, even as small droplets. TMH-Fc, on the other hand, could make its way through general body surface for first instar *Artemia*. This is a promising area for further research using ferrocene derivatives and associated biological effects in other ecotoxicological models at different stages of development.

**Fig 4:**
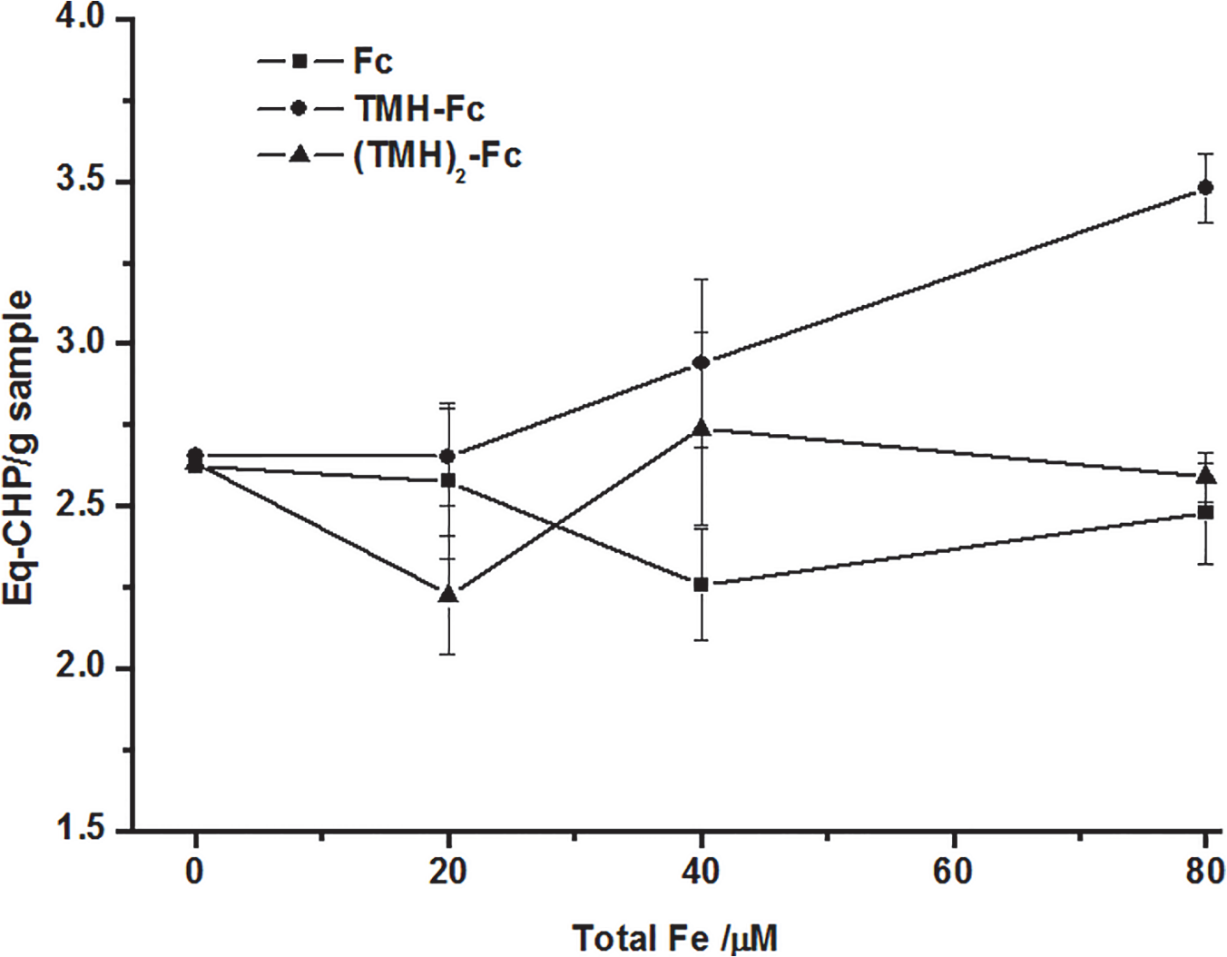
Lipid peroxidation (measured as CHP equivalents per gram of *A*. *salina*) after the treatment with ferrocene derivatives.

As a means to better understand the mechanisms of toxicity, we determined the amount of lipid peroxidation caused by the Fc derivatives in stage 1 *A*. *salina* (Fig 4). In agreement with toxicity tests, only TMH-Fc induced a significant increase in lipid peroxidation (3.5 Eg-CHP) at a concentration (80 μM) similar to the LD50, indicating that this could be one of the possible mechanisms to explain the higher toxicity of TMH-Fc to *A*. *salina*.

The absence of a clear relationship between aqueous stability and toxicity indicates that caution must be observed when assaying (eco)toxicity of metal complexes. Ecotoxicological models (Free Ion Availability, Biotic Ligand Model) [20,21] assume equilibrium conditions and predict that metal toxicity will generally be directly related to the amount of free metal available. However, ours and numerous other results seem to contradict this assumption, as organisms may actively internalize metals complexed with specific ligands, passive diffusion of non-charged, lipophilic metal compounds may occur, or finally organisms may have promiscuous binding sites that are used by different metal species [20,21]. This last possibility is of extreme importance when the metallodrugs are based on iron, an essential element for animals.

## Conclusions

Different probes with distinct binding sites have access to different species of chelatable iron. Ferrocene derivatives metallodrugs, studied here, have lower stability and considerable higher percentage of redox-active iron, when compared to the polymer-coated iron metallodrugs of commercial use. Therefore, the toxicity of the iron supplements of commercial origin was negligible; however, the mono-substituted ferrocene derivative (TMH-Fc) displayed high toxicity to stage 1 Artemia, possibly due to increased absorption through general body surface area followed by higher lipid peroxidation. Fc, on the other hand, was more toxic to adult Artemia. We suggest that ecotoxicity depends more on metal speciation (in this case, iron) present in the metallodrug, than on the total or free amount of metal present. Future studies with discarded metallodrugs should consider the chemical speciation of the metal present in the composition of the drug.
